# Introduction and welcome

**DOI:** 10.1186/1476-9255-12-S1-O1

**Published:** 2015-04-16

**Authors:** Raif Yuecel

**Affiliations:** 1 Iain Fraser Cytometry Centre, Institute of Medical Sciences, School of Medicine & Dentistry, University of Aberdeen, Foresterhill, AB25 2ZD, Aberdeen, UK

## 

It was my greatest pleasure to present the 1st Annual meeting of the newly formed Scottish Society of Cytomics (SSC) in Aberdeen, UK as host and SSC chair. The aim of establishing a new technological science group in Scotland is to bring scientists in the field of cytomics from cross technology centres together. Why Cytomics? Actually, the theme of our first SSC meeting already provides the answer - Translational Cytometry from Bench to Bedside- relates to the future of this society and it reflects our shared passion for cellular systems. Cytometry is growing as a diagnostic and monitoring technology in biological research and in the clinical laboratory and therefore it is useful to establish a forum to exchange information, to present research data and to explore new applications of the rapidly expanding technologies of imaging and flow cytometry. To better understand and further explore the cellular systems, the merging and bundling of technologies is necessary. Single technology can’t provide the ultimate solution. Therefore, CYTOMICS, which analyzes the functional relationship between cell systems and networks using the “omics” technologies (e.g., Genomics, Proteomics), will give a comprehensive view of a disease. The analysis of already existing data from scientific studies or routine diagnostic procedures makes cytomics a valuable tool for studying diseases, that will be of immediate value in translational medicine.

The Scottish Society of Cytomics was formed with the intention of providing information related to the art of cytometry (flow and imaging) including its cross technology applications to genomics, proteomics, *in vivo* imaging and other core related areas between the research organisations in Scotland and Northern England. We believe that SSC will be the ideal forum for users of cytometry and omics systems to get together, share expertise and innovation, discuss pitfalls, successes and ideas for the future of translational cytomics. It was a great privilege for me to announce the exciting line up of talks, workshop and exhibitions. SSC 2014 brought together top scientists from various disciplines, and international renowned keynote speakers. Further to the oral presentations we were happy with the quality of abstracts submitted by motivated scientists. This poster presentation resulted in poster awards to recognize the excellent research. I would like to take the opportunity to acknowledge the support and generosity of the SSC 2014 sponsors in making this meeting a success. Our special thank goes to Becton Dickinson, Sony, BioStatus and Miltenyi Biotec for their extra donations and contributions. In conclusion this conference brought the rich and rewarding experiences for all of us in science (>150 attendees), and helped to develop friendships. We hope that our Society will be a platform for future success in cytomics research and invite you to participate in these activities. Welcome to our newly formed Scottish Society of Cytomics that hopes to foster a strong network for this scientific community.

